# Do Parenting Styles Influence Mental Toughness and Sportsmanship in Young Athletes? A Structural Equation Modelling Approach

**DOI:** 10.5114/jhk/188541

**Published:** 2024-12-06

**Authors:** Marta Vega-Díaz, Higinio González-García

**Affiliations:** 1 Faculty of Education, Universidad Internacional de La Rioja (UNIR), Logroño, Spain.; 2 TECNODEF Research Group, Department of Physical Education and Health, Faculty of Education, Universidad Internacional de La Rioja (UNIR), La Rioja, Spain.

**Keywords:** parenting, sports, competitors, coaching, youngsters

## Abstract

The goal of this research was to evaluate whether there are relationships between parenting styles, mental toughness, and sportsmanship in athletes. The sample was made up of 201 Spanish adolescent athletes (Mage = 15.30; SD = 1.82; 110 men and 91 women) who completed the Child-Parental Acceptance-Rejection Questionnaire, the Mental Toughness Inventory, and the Spanish version of the Multidimensional Sportsmanship Questionnaire. Structural equation modeling was used to examine the relationships among the studied variables. The results revealed that maternal and paternal love/affection significantly positively predicted mental toughness. Likewise, the results showed that paternal hostility/aggression and indifference/neglect significantly positively predicted fair play. In conclusion, given the connection between perceived parental education, psychological variables (mental toughness), and sports ethics principles (sportsmanship) of athletes, programs that train athletes' cognitive and axiological skills should not ignore the parental role. Hence, an attempt could be made to encourage athletes to compete when feeling mentally strong and respecting the ethical values of sportsmanship.

## Introduction

Mental toughness has been a highly studied variable in different contexts and sports modalities (Cowden et al., 2020; Liew et al., 2019; Powell and Myers, 2017; Pronczuk et al., 2023, 2024; Rintaugu et al., 2022; Skalski et al., 2024). Likewise, the social vision of sportsmanship has been widely addressed in sports psychology (Burgueño and Medina-Casaubón, 2020; Charles, 2011; Sáenz et al., 2013). Mental toughness can be conditioned by external agents such as coaches and parents (Guszkowska and Wójcik, 2021), as is the case with sportsmanship (González et al., 2010). However, given that the family is a primary agent that influences the mental toughness of children (Qi et al., 2023) and parents are crucial for the transmission of positive values regarding sportsmanship (Danioni et al., 2017), this project will direct the focus to the figure of parents through parenting styles (PSs).

In particular, PSs are the educational behaviors offered by parents to their children (Darling and Steinberg, 1993). In this research, to examine PSs, the parental acceptance-rejection theory (PARTheory; Rohner, 1986) was followed. This is a pan-culturally accepted socialization theory that shows that parents influence their children's psychological adjustment and behaviors depending on their acceptance and rejection (Rohner and Lansford, 2017). The PARTheory Spanish version includes five domains: love/affection, hostility/aggression, indifference/neglect, undifferentiated rejection, and control (Del-Barrio et al., 2014). Love/affection refers to interest and parental-filial warmth. Hostility/aggression is the perception of parents as physical or verbal aggressors. Indifference/neglect examines the degree of attention and care parents give their children. Undifferentiated rejection explores the disaffection expressed by parents. Finally, control assesses how parents interfere with their children's behaviors.

Although the mental toughness (MT) variable has been studied previously in the sports field (Poulus et al., 2020; Reche-García et al., 2022), there is little research specifically addressing the topic of PSs and MT. MT refers to the ability to outperform an opponent under pressure and remain focused on and confident in a task (Jones et al., 2002). There are eight characteristics that define a mentally strong athlete: the ability to achieve goals, self-control when performing a task, use of emotions in the way one wants, continually striving for success, use of knowledge to achieve goals, overcoming adversity, use of skills in challenges, and finding the positive of the situation (Gucciardi et al., 2015). The aforementioned characteristics are vital in sports because people who possess them have a higher ability to handle pressure, control themselves, and work toward their goals, which means they have higher MT (Jones, 2008).

Some of the literature postulates that parents providing love/affection have a positive relationship with high levels of MT (Zhang and Li, 2019). Rathi and Singh (2017) found that hostility/aggression was not related to MT. Parental indifference/neglect relates to childhood psychological maladjustment and negative perceptions of self-efficacy (Khaleque, 2014). A low perception of self-efficacy could negatively affect perceived control in different situations that may arise during competition (Bandura, 2006). Consequently, it would be negatively related to MT. Parental undifferentiated rejection is related to poor psychological adjustment (Putnick et al., 2019), which predicts the use of dysfunctional coping strategies (Cofini et al., 2015). People with dysfunctional coping strategies perceive adverse situations as threats, not as challenges to overcome, and their MT is not high (Nicholls et al., 2012). Wood et al. (2003) found that parental undifferentiated rejection increased children's anxiety levels, which could inhibit perceptions of control in different situations (Šrol et al., 2021). If the low perception of control is transferred to sport, it will weaken MT. Finally, Zhang and Li (2019) showed that low parental control enhanced MT.

Despite previous studies addressing sportsmanship (Cosma et al., 2021; Núñez et al., 2009; Özsari et al., 2023), this may be one of the first works to examine the relationship between PSs and sportsmanship in athletes. This aspect is worth considering because it will allow us to determine whether some behaviors of athletes in sports could be linked to PSs. Athletes must compete with sportsmanship, an ethical ideal that becomes a reality when players respect the game's rules and their opponents (Iturbide-Luquin and Elosua-Oliden, 2017), and which is intrinsically associated with the concept of value (Sallis et al., 2012). Iturbide-Luquin and Elosua-Oliden (2017) postulated five values that make sportsmanship: enjoyment, respect, commitment, fair play, and participation. Enjoyment is the feeling of recreation perceived by participating in sports. Respect is tolerance toward sports and peers. Commitment refers to cooperation with colleagues and pursuing the scope of excellence. Fair play implies compliance with social conventions and rules. Participation is the genuine desire to put in the effort at the expense of knowing what will be lost during competition. As a novelty, this study could allow to determine if maternal and paternal PSs are related to the sportsmanship of athletes, which would serve to indicate the need of an intervention if a PS is related to unethical values during a competition.

Some research postulates a typology of parents whose PS is functional because it provides love/affection and moderate control (Mandara, 2006), and helps children not engage in deviant behaviors. These children will not violate social principles and values (Clinard and Meier, 2007); thus, they should respect values and fair play. On the other hand, there are different types of dysfunctional PSs. The first dysfunctional PS is adopted by parents that exercise hostility/aggression (physical punishment) and excessive control (Ang and Goh, 2006). These parents never negotiate the rules with their children: they impose their decisions (Berger, 2001). Therefore, these parents hinder their children’s choice of the sport to develop (Pelegrín and Garcés de los Fayos, 2004), which could have a negative relationship with the positive assessment of participation and enjoyment (González-García, 2017). The second case of dysfunctional PSs reflects parents who do not care, are not concerned or do not supervise their children (indifference, neglect, and lack of control) (Kaliadem, 2005). This kind of parents hinders children from being mature and taking responsibility for their actions (Igbo and Ihejiene, 2014), which could be negatively related to the value of commitment. Kaliadem (2005) states that dysfunctional PSs lead children to adopt deviant behaviors. Therefore, it does not seem unreasonable to consider that hostility/aggression, indifference/neglect, and undifferentiated rejection positively relate to low respect and fair play values. Finally, a lack of parental control hinders parents from intervening in their children's misbehavior, which does not favor the children’s change in attitude over time (Igbo and Ihejiene, 2014).

Previous studies have examined MT (Poulus et al., 2020; Reche-García et al., 2022) and sportsmanship (Cosma et al., 2021; Özsari et al., 2023) in the sports field, but it seems that they ignored the relationship with PSs. Our study goes a step further in identifying the relationship between the most functional dimensions of PSs (maternal and paternal separately) and MT and sportsmanship of athletes. It would be very relevant to design intervention programs, considering PSs, to ensure that athletes compete feeling safe, capable, and respecting the ethical values of sport. Previous works found that love/affection was positively related to MT (Zhang and Li, 2019), while hostility/aggression did not condition this variable (Rathi and Singh, 2017). Indifference/neglect (Khaleque, 2014) and undifferentiated rejection (Putnick et al., 2019) were related to variables that weakened MT, and low control enhanced MT (Zhang and Li, 2019). On the other hand, parental love/affection and control were unrelated to deviant behaviors (Clinard and Meier, 2007), or what would relate here to respect and fair play. Parental hostility/aggression and excessive control facilitate ignoring children's preferences, diminishing children’s participation and enjoyment (González-García, 2017). Indifference, neglect, and lack of control promote the immaturity of children (low commitment). Therefore, the goal of this research was to determine whether there is a relationship between PSs, MT, and sportsmanship in athletes. Considering these previous studies, research hypotheses were as follows: (a) Perception of parental love/affection and moderate control will be positively related to children’s MT; perception of indifference/neglect and undifferentiated rejection will be negatively related to children’s MT; perception of parental love/affection will be positively related to children’s sportsmanship; and perception of hostility/aggression, indifference /neglect, undifferentiated rejection, and lack of control will be negatively related to children’s sportsmanship. (b) Perception of a lack of parental love/affection and moderate control will be negatively related to children’s MT; perception of a lack of indifference/neglect and undifferentiated rejection will be positively related to children’s MT; perception of a lack of parental love/affection will be negatively related to children’s sportsmanship; and perception of a lack of hostility /aggression, indifference/neglect, undifferentiated rejection, and lack of control will be positively related to children’s sportsmanship.

## Methods

### Participants

The participants included 201 Spanish athletes aged between 12 and 18 years old (M *age* = 15.30; *SD* = 1.82; 110 male teenagers and 91 female teenagers). A minority worked part-time or full-time (*n* = 11) or were unemployed (*n* = 2), while most were students (*n* = 188). On the other hand, of the 201 athletes, 69 were associated to federations and 132 were not associated. Some of the participants were currently competing at the local (*n* = 35), regional (*n* = 24), national (*n* = 13), and international (*n* = 7) level. In contrast, the other participants did not compete at any recognized level, but participated in competitive sports. Some of the participants revealed local successes (*n =* 46), regional successes (*n =* 25), national successes (*n =* 28), and international successes (*n =* 14). The sample selection was random. As inclusion criteria, we focused on the Spanish adolescent population between 12 and 18 years old who practiced competitive sports and were raised by their mother and father. The age requirement was established because we wanted to find out whether a relationship existed between PSs, MT, and sportsmanship in the competitive sports field in young people.

### Measures

To evaluate perceived PSs, the Spanish version of the Child-Parental Acceptance-Rejection Questionnaire (Child PARQ/Control; Rohner, 2005) was used. The adaptation of the Spanish version consists of 29 items (Del-Barrio et al., 2014). The Child PARQ/Control Questions are answered by sons and daughters to know their perception of the maternal and paternal PS. Besides, although the questions related to maternal and paternal figures are identical, the offspring answered them separately to collect information from the mother and the father. The ChildPARQ/Control scale is used to measure love/affection (mother, α = 0.87; father, α = 0.90; eight items, e.g., “My mother/father loves me and needs me”), hostility/aggression (mother, α = 0.90; father, α = 0.92; six items, e.g., “My father/mother gets angry and hurts my feelings”), indifference/neglect (mother, α = 0.82; father, α = 0.85; six items, e.g., “My father/mother ignores me”), undifferentiated/rejection (mother, α = 0.87; father, α = 0.89; four items, e.g., “My father/mother really does not love me”), and control (mother, α = 0.60; father, α = 0.60; five items, e.g., “My father/mother wants to control everything I do”). The responses correspond to a Likert-type scale ranging from 1 (almost never true) to 4 (almost always true).

The Mental Toughness Inventory (MTI; Gucciardi et al., 2015) was administered to measure MT. The MTI measures psychological attributes that enable one to overcome challenges in the face of adversity and are essential in determining success in sport. It consists of eight items that measure MT (α = 0.88, eight items, e.g., “I believe in my ability to achieve my goals”). The responses correspond to a Likert-type scale ranging from 1 (false, 100% of the time) up to 7 (true, 100% of the time).

To evaluate athletes' sportsmanship, the Spanish version of the Multidimensional Sportsmanship Questionnaire (MSQ; Iturbide-Luquin and Elosua-Oliden, 2017) was administered. It consists of 21 items that measure participation (α = 0.72, four items, e.g., “I don't mind losing if I'm having fun”), enjoyment (α = 0.91, five items, e.g., “I play to feel good”), fair play (α = 0.78, four items, e.g., “I react to provocation”), respect (α = 0.88, four items, e.g., “I show respect toward opponents”), and commitment (α = 0.88, four items, e.g., “I respect the rules of the game”). The responses correspond to a Likert-type scale ranging from 1 (never) to 5 (always).

### Design and Procedures

The study complied with the ethical guidelines established by the American Psychology Association in its seventh edition (APA 7) (American Psychology Association, 2020) and was approved by the ethics committee of the Universidad Internacional de la Rioja (approval code: 074/2022; approval date: 11 October 2022). Moreover, anonymity was preserved, and the principles of the Declaration of Helsinki were followed. The study sample participants were informed online of the research purpose through a publication posted on a group comprising young athletes. Interested individuals responded to the post and contacted the researchers. As participants were minors, a consent form was sent to the parents before the study began. When the parents gave their consent for their children to participate in the research and sent the signed agreement, they received the link for the children’s survey. The children responded to the form through the Google Forms platform. The questionnaire was divided into three different sections (each of the instruments used was included in a section and, prior to the beginning of the questions, it was explained how participants had to complete the questionnaire in order to carry them out). In addition, in the event of any concern, participants had the email addresses of researchers, whom they could contact at any time. Subsequently, participants answered the questionnaires with absolute freedom through the link in their email. When a participant completed the questionnaire, the data were automatically stored in the application and the new information was visible to the researcher. The data were automatically recorded in Microsoft Excel 365 and later exported to Mplus for statistical analysis.

### Statistical Analysis

All the analyses were conducted using Mplus Version 7.3. To evaluate the relationships among the variables, structural equation modeling (SEM) was performed. SEM refers to statistical models that allow the exploration of relationships among variables and include latent variables in the same model. This approach avoids type I errors compared to independent tests of each relationship. In accordance with Anderson and Gerbing (1988), two-step modeling was performed, consisting of the measurement model (step 1) and the structural model (step 2). For the measurement model step, the correlated model was calculated. Then, items were averaged to create parcels (Little et al., 2002) of PSs (love/affection, hostility/aggression, indifference/neglect, undifferentiated rejection, and control behavior), MT, and sportsmanship (participation, enjoyment, fair play, respect, and commitment). For instance, the first, third, fifth, and seventh items of love/affection were averaged to create one of the two parcels. Later, the second, fourth, sixth, and eighth items of love/affection were averaged to create the other parcel. Likewise, the first, third, and fifth items of hostility/aggression and indifference/neglect were averaged to create one of the two parcels. Then, the second, fourth, and sixth items of the aforementioned variables were averaged to create the other parcel.

For undifferentiated rejection, the first and third items were averaged to create one parcel; the operation was then repeated with the second and fourth items. Finally, for control, two parcels were made. For the first, the first and third items were averaged, while for the second, the second, the fourth, and the fifth items were averaged. Regarding MT, two parcels of four items were built. The first, third, fifth, and seventh items were averaged for the first. For the second parcel, the rest of the items were averaged. Finally, two parcels were built for participation, fair play, respect, and commitment. The first and third items were averaged for the first, and the second and fourth items were averaged for the second. The enjoyment variable was divided into two parcels: the first and third items were averaged for the first parcel, while the second, fourth, and fifth items were averaged for the second parcel.

The data were screened for multicollinearity of scales, because the collinearity of predictors can unduly influence the results of multilevel analyses in potentially unfavorable ways (Vacher et al., 2017). Multicollinearity (Pearson's correlation coefficient) can take values between −1 and 1 (*r* = 1 perfect correlation; 0.8 < *r* < 1 very high correlation; 0.6 < *r* < 0.8 high correlation; 0.4 < *r* < 0.6 low correlation; 0 < *r* < 0.2 very low correlation; and *r* = 0 null correlation).

We used a combination of indices to achieve a comprehensive evaluation of fit (Hu and Bentler, 1999), including the chi-square (χ^2^), the Comparative fit index (CFI), the Tucker-Lewis index (TLI), the root mean square error of approximation (RMSEA) and its confidence interval (90% CI). The CFI and TLI of 0.90 and 0.95 reflect acceptable and excellent fits, respectively, whereas the RMSEA of less than 0.06 and 0.08 reflect close and reasonable fits, respectively (Can de Schoot et al., 2012).

## Results

Descriptive statistics of the study variables are presented in Table 1. Low to high scores of PSs (between −0.20 and 0.91) were reported by participants (i.e., love/affection, hostility/aggression, indifference/neglect, undifferentiated/rejection, and control). Regarding MT, low to medium scores (between −0.28 and 0.40) were reported by participants. With respect to sportsmanship (participation, enjoyment, fair play, respect, and commitment), participants reported medium to high scores (between 0.28 and 0.84). Besides, low to average scores (from −0.19 to 0.40) were found between PS and MT. Finally, low to average scores between PS and sportsmanship (from −0.25 to 0.49) were found. No collinearity was detected between PS and MT and between PS and sportsmanship.

**Table 1:** Descriptive statistics and correlations among the studied variables.

	1	2	3	4	5	6	7	8	9	10	11	12	13	14	15	16
1.	-															
2.	−0.20^**^	-														
3.	−0.42^**^	0.80^**^	-													
4.	−0.23^**^	0.91^**^	0.83^**^	-												
5.	0.06	0.64^**^	0.49^**^	0.58^**^	-											
6.	0.43^**^	0.09	0.05	0.07	0.22^**^	-										
7.	−0.08	0.75^**^	0.64^*^	0.77^**^	0.45^**^	0.06	-									
8.	−0.20	−0.60^**^	0.63^**^	0.63^**^	0.35^**^	−0.27^**^	0.73^**^	-								
9.	−0.01	0.71^**^	0.61^**^	0.72^**^	0.44^**^	0.22^**^	0.83^**^	0.64^**^	-							
10.	0.05	0.47^**^	0.32	0.47^**^	0.52^**^	0.23^**^	0.56^**^	0.41^**^	0.54^**^	-						
11.	0.40^**^	−0.20^**^	−0.28^**^	−0.19^**^	−0.06	0.38^**^	−0.16^*^	−0.20	−0.05	−0.08	-					
12.	0.22^**^	−0.01	−0.04	0.01	0.09	0.25^**^	0.05	−0.03	0.06	−0.05	−0.08	-				
13.	0.29^**^	−0.26^**^	−0.31^**^	−0.27^**^	−0.06	0.08	−0.25^**^	−0.17	−0.20^*^	−0.13^*^	0.41^**^	0.47^**^	-			
14.	0.04	0.25^**^	0.20^**^	0.24^**^	0.20^*^	0.00	0.26^**^	0.23^**^	0.22^**^	0.11	0.13	0.32^**^	0.39^**^	-		
15.	0.22	−0.24^**^	−0.26^**^	−0.23^**^	−0.13	0.13	−0.17^*^	−0.10	−0.09	−0.06	0.50^*^	0.50^**^	0.71^**^	0.31^**^	-	
16.	0.30^**^	−0.22^**^	−0.26^**^	−0.22^**^	−0.06	0.12	−0.19^**^	−0.13	−0.11	−0.04	0.49^**^	0.45^**^	0.80^**^	0.28^**^	0.84^**^	-
*M*	3.24	1.75	1.99	1.75	2.45	2.86	1.73	2.08	2.12	2.31	5.61	3.33	4.10	4.12	3.91	4.08
*SD*	0.66	0.82	0.71	0.84	0.74	0.66	0.85	0.75	0.67	0.62	0.96	0.83	0.88	0.99	0.93	0.86
*S*	-1.10	1.07	0.47	1.11	0.29	−0.81	0.90	0.5	0.83	0.00	−0.43	−0.13	−0.62	−0.04	−0.74	−0.84
*K*	0.98	0.20	−0.74	0.23	−0.69	0.25	−0.20	−0.90	0.26	−0.78	−0.29	0.40	−0.55	0.27	0.29	−0.38

Note. * p < 0.05; ** p < 0.01; M : Mean; S : Skewness; K: Kurtosis; 1. Maternal Love/Affection; 2. Maternal Hostility/Aggression; 3. Maternal Indifference/Neglect; 4. Maternal Undifferentiated Rejection; 5. Maternal Control; 6. Paternal Love/Affection; 7. Paternal Hostility/Aggression; 8. Paternal Indifference/Neglect; 9. Paternal Undifferentiated Rejection; 10. Paternal Control; 11. Mental Toughness; 12. Participation; 13. Enjoyment; 14. Fair play; 15. Respect; 16. Commitment

### Preliminary Analyses

The goodness-of-fit indexes of the general model are presented in Table 2. The model was acceptable as the indexes ranged appropriately (CFI = 0.95; TLI = 0.93; RMSEA = 0.065).

**Table 2:** Fit Indices for the Measurement Models.

*Model*	X^2^	CFI	TLI	RMSEA	90% CI RMSEA	AIC	BIC	ABIC
General	5266.24	0.953	0.932	0.057	0.049–0.065	11386.39	12099.90	11415.58

Note. CFI = comparative fit index; TLI = Tucker-Lewis index; RMSEA = root-mean-square error of approximation; CI = confidence interval; AIC = Akaike’s information criterion; BIC = Bayesian information criteria; ABIC = sample-size adjusted BIC

### Standardized Estimates for the Structural Model

**Figure 1:**
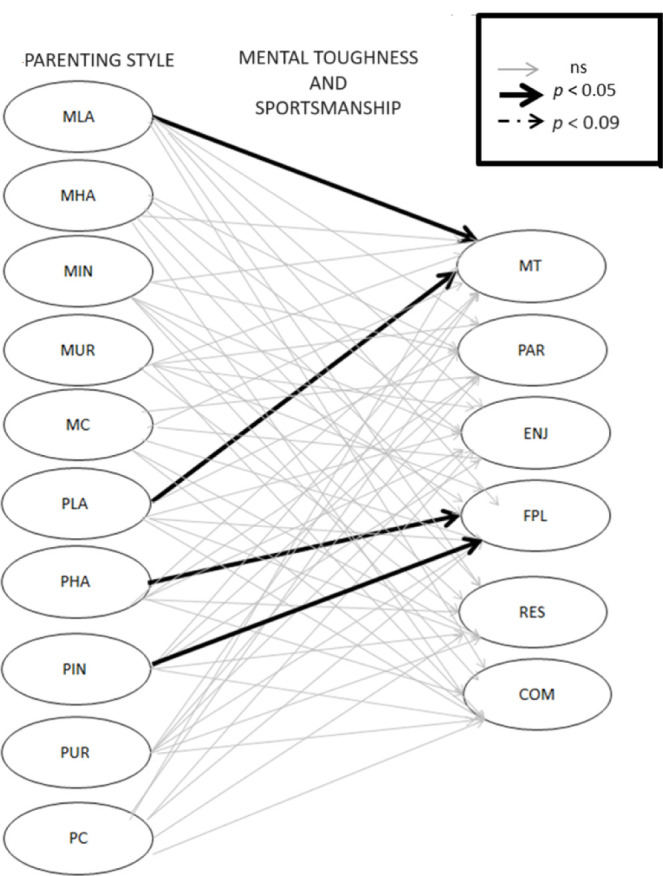
Results of the structural model of the partial least square – path modelling in general model. *MLA, Maternal Love/Affection; MHA, Maternal Hostility/Aggression; MIN, Maternal Indifference/Neglect; MUR, Maternal Undifferentiated Rejection; MC, Maternal Control; PLA, Paternal Love/Affection; PHA, Paternal Hostility/Aggression; PIN, Paternal Indifference/Neglect; PUR, Paternal Undifferentiated Rejection; PC, Paternal Control; MT, Mental Toughness; PAR, Participation; ENJ, Enjoyment; FPL, Fair Play; RES, Respect; COM, Commitment*.

Results of the relationships among the variables are summarized in Table 3 and Figure 1. Results revealed that maternal love/affection significantly positively predicted MT (*β* = 0.39; *p* < 0.01). Likewise, results revealed that paternal love/affection significantly positively predicted MT (*β* = 0.46; *p* < 0.01). Finally, results revealed that paternal hostility/aggression significantly positively predicted fair play (*β* = 0.39; *p* < 0.05), and that indifference/neglect significantly positively predicted fair play (*β* = 0.32; *p* < 0.05).

**Table 3:** Standardized Estimates for the Structural Model: parenting styles, mental toughness, and sportsmanship.

Dependent variables of the general model	Independent Variables	Estimate (*β*)	S.E.	*p*
Maternal Love/Affection	Mental toughness	0.39	0.13	0.00**
Maternal Hostility/Aggression	Mental toughness	−0.10	0.34	0.74
Maternal Indifference/Neglect	Mental toughness	−0.17	0.35	0.63
Maternal Undifferentiated/Rejection	Mental toughness	−0.10	0.35	0.76
Maternal Control	Mental toughness	0.08	0.49	0.87
Paternal Love/Affection	Mental toughness	0.46	0.16	0.00**
Paternal Hostility/Aggression	Mental toughness	−0.10	0.28	0.70
Paternal Indifference/Neglect	Mental toughness	−0.14	0.19	0.45
Paternal Undifferentiated/Rejection	Mental toughness	−0.08	0.25	0.73
Paternal Control	Mental toughness	0.13	0.48	0.78
Maternal Love/Affection	Participation	−0.07	1.01	0.94
Maternal Love/Affection	Enjoyment	0.87	3.83	0.81
Maternal Love/Affection	Fair play	−0.07	0.12	0.52
Maternal Love/Affection	Respect	0.82	5.95	0.89
Maternal Love/Affection	Commitment	−1.45	8.70	0.86
Maternal Hostility/Aggression	Participation	−0.39	3.21	0.90
Maternal Hostility/Aggression	Enjoyment	1.79	12.08	0.88
Maternal Hostility/Aggression	Fair play	0.41	0.29	0.16
Maternal Hostility/Aggression	Respect	3.31	18.53	0.85
Maternal Hostility/Aggression	Commitment	−4.87	27.42	0.85
Maternal Indifference/Neglect	Participation	−0.43	3.39	0.90
Maternal Indifference/Neglect	Enjoyment	1.87	12.77	0.88
Maternal Indifference/Neglect	Fair play	0.43	0.31	0.16
Maternal Indifference/Neglect	Respect	3.60	19.55	0.85
Maternal Indifference/Neglect	Commitment	−5.19	28.75	0.85
Maternal Undifferentiated/Rejection	Participation	−0.37	3.33	0.91
Maternal Undifferentiated/Rejection	Enjoyment	1.78	12.52	0.88
Maternal Undifferentiated/Rejection	Fair play	0.41	0.30	0.17
Maternal Undifferentiated/Rejection	Respect	3.47	19.19	0.85
Maternal Undifferentiated/Rejection	Commitment	−5.05	28.20	0.85
Maternal Control	Participation	−0.45	4.79	0.92
Maternal Control	Enjoyment	3.05	17.87	0.86
Maternal Control	Fair play	0.31	0.45	0.49
Maternal Control	Respect	4.63	27.55	0.86
Maternal Control	Commitment	−7.22	40.39	0.85
Paternal Love/Affection	Participation	−0.06	1.62	0.97
Paternal Love/Affection	Enjoyment	1.02	6.09	0.86
Paternal Love/Affection	Fair play	−0.11	0.17	0.51
Paternal Love/Affection	Respect	1.49	9.42	0.87
Paternal Love/Affection	Commitment	−2.41	13.80	0.86
Paternal Hostility/Aggression	Participation	−0.23	2.73	0.93
Paternal Hostility/Aggression	Enjoyment	1.25	10.20	0.90
Paternal Hostility/Aggression	Fair play	0.39	0.24	0.00**
Paternal Hostility/Aggression	Respect	−0.16	0.42	0.09
Paternal Hostility/Aggression	Commitment	2.87	15.57	0.85
Paternal Indifference/Neglect	Participation	−0.20	1.58	0.89
Paternal Indifference/Neglect	Enjoyment	0.68	6.05	0.91
Paternal Indifference/Neglect	Fair play	0.32	0.15	0.03*
Paternal Indifference/Neglect	Respect	1.81	9.13	0.84
Paternal Indifference/Neglect	Commitment	−2.38	13.50	0.86
Paternal Undifferentiated/Rejection	Participation	−0.21	2.44	0.93
Paternal Undifferentiated/Rejection	Enjoyment	0.99	9.13	0.91
Paternal Undifferentiated/Rejection	Fair play	0.36	0.22	0.10
Paternal Undifferentiated/Rejection	Respect	2.62	13.90	0.85
Paternal Undifferentiated/Rejection	Commitment	−3.56	20.47	0.86
Paternal Control	Participation	−0.74	4.72	0.87
Paternal Control	Enjoyment	2.64	17.75	0.88
Paternal Control	Fair play	0.37	0.43	0.39
Paternal Control	Respect	5.03	27.21	0.85
Paternal Control	Commitment	−7.02	40.00	0.86

Note. p < 0.01**; p < 0.05*

## Discussion

This research aimed to determine whether there is a relationship among parenting styles, mental toughness, and sportsmanship in athletes. The results of the study confirmed a relationship between PSs and MT. Particularly, maternal and paternal love/affection positively predicted MT. Previously, Zhang and Li (2019) postulated that parents’ provision of love/affection had a positive relationship with high levels of MT. This occurs because parental acceptance (manifested by love/affection) is consistently related to children's psychological adjustment (Rohner, 2021; Rohner and Lansford, 2017). Therefore, parents who express love/affection enhance their children's ability to achieve goals, their self-control when performing a task, their ability to use emotions in the way they want to, their capacity to strive for success continually, their ability to use knowledge to achieve goals, their capacity to overcome adversity, and their ability to use skills in challenges and find the positive in different situations. In conclusion, they have the characteristics of a mentally strong person (Gucciardi et al., 2015). At the same time, MT often leads to sporting success (Cowden, 2017; Drees and Mack, 2012; Jooste et al., 2023). As such, it increases the possibility of personal growth in sports and the chance of reaching the elite (Rodríguez, 2016). However, we may also find research in the literature where excessive protectionism (González-García et al., 2018) showed opposite results in terms of achieving success.

Regarding the relationship between PS and sportsmanship, it was found that paternal hostility/aggression positively predicted fair play. Kaliadem (2005) stated that this dysfunctional PS led children to adopt deviant behaviors. Therefore, a positive relationship between paternal hostility/aggression and fair play was not expected. In the same way, we found that indifference/neglect significantly positively predicted fair play. Previous works have found parents who indicated that the children’s experiences did not matter (Westphal et al., 2016), and they utilized aversive behaviors (e.g., yelling, negative physical touch) more quickly than non-maltreating parents (Wilson et al., 2008). Hence, Kaliadem (2005) considered that this PS led children to adopt deviant behavior. Therefore, it was expected that paternal indifference/neglect would be related to low fair play values. Given that the results for parental hostility/aggression and indifference/neglect are contrary to those expected, a possible explanation is that parents who are more concerned about their jobs and careers transfer the obligations of their children's education to the coaches (Witkowski et al., 2016). Therefore, the role of a coach (a sports educator with pedagogical skills) is essential to configure the establishment of appropriate interpersonal relationships among athletes that will avoid the adoption of deviant behaviors (Witkowski et al., 2016). However, we cannot ignore that the educational work of athletes can also be inadequate. For example, previous research has confirmed the existence of coaches that make use of verbal aggression (teasing, threats, profanity) (Bekiari and Syrmpas, 2015). Similarly, reference is made to negligent athletes who use sexual abuse (Parent et al., 2016), fail to meet the reasonable standard of care expected of them (which can lead to injuries in athletes) (Partington, 2015), or do not respect the relevant codes of conduct.

Bandura's social learning theory postulates that a person's socialization results from observation and perceived cultural modeling (Bandura, 1978). In other words, socialization can shape people's behavior in particular directions and with specific attitudes. Given that fair play (in addition to respecting the rules) implies respect for teammates and opponents, and that behavior is learned by modeling (Bandura, 1978), it would be expected that hostility/aggression and indifference/neglect favor low fair play values. It therefore seems likely that coaches who worked with athletes from our sample applied a functional pedagogical educational model, capable of counteracting the perceived dysfunctional PS variables in sport (Witkowski et al., 2016). On the other hand, fair play also depends on psychological, sociocultural, and moral reasoning factors (Mujica et al., 2018). Perhaps sociocultural factors (social acceptance of fair play as a positive ideal) and the morality of athletes in this research had greater weight in this value of sportsmanship than the role of the parents themselves. Thus, athletes may not want to win by all means, but prefer to achieve victory without using cheats.

Among the limitations of this study, it should be highlighted that the variables examined were evaluated with Spanish minors, practitioners of competitive sports, and raised by both the mother and the father. Nevertheless, the study sample included a specific number of participants who competed at a local, regional, national, and international level (which will not coincide with other samples). Therefore, the results may not be generalizable to people of other nationalities, different age ranges, non-competitive sports practitioners, and sedentary people. In addition, the methodology used in the analysis was based exclusively on data from the self-report questionnaire, which could have a slight bias in the results (desire to perceive another PS or to be more mentally strong). Therefore, in future research, it would be advisable to include samples of other nationalities, different age groups, athletes who participate in non-competitive sports, and sedentary people. This will allow to evaluate whether the most functional variables of PSs have the same relationship with MT and sportsmanship. On the other hand, other variables, such as the motivational climate offered by parents, should be examined. A priori, the motivational climate that induces learning and fun during sports practice should generate greater MT and sportsmanship.

As practical implications, this work conveys that parental expressions of affection (hugs, kisses, encouragement) have a positive relationship with MT. As such, mothers and fathers should not repress love/affection since it will enhance children's mental toughness. In addition, this research reflects that parental indifference/neglect is not necessarily related to values opposite to fair play. This is because sportsmanship is influenced by external factors such as coaches, the sociocultural climate, moral climate, etc. In training of athletes, a multitude of factors, such as contextual, psychological, and moral, should be considered, since there are connections among them that significantly influence participants' behavior during competitions.

## Conclusions

In conclusion, MT and sportsmanship have a relationship with PSs. Parents should reflect on the parenting pattern they offer during childhood since the PS is related to the children's psychological dimension (MT) and ethics (sportsmanship). Therefore, mothers and fathers who want their children to have good psychological adjustment (mediated by MT) and practice sports should use functional variables of PSs (e.g., love/affection) from early childhood. Parents are not the only external factors related to the morality of their children (coaches, peers, media, etc. will also have an influence). When parents do not spend much time with their children, this may enhance the influence of those external factors. In such a case, the sportsmanship of athletes may be more conditioned by coaches than by their own families.

In addition, a specific number of athletes who competed at different levels participated in this study, which hinders the generalizability of the results to a wider population. This suggests the importance of replicating this study in other populations to examine if the results evolve in the same direction as in the current research. Despite this, given the existence of a relationship among PS, MT, and sportsmanship, programs that train athletes' cognitive abilities should consider the important role played by parents to create mentally strong athletes with adequate moral values.
